# What Do Case Studies Tell Us About Addictions and Psychiatric Comorbidities? A Survival Story: The necessity for a transdiagnostic and holistic approach

**DOI:** 10.1192/j.eurpsy.2024.862

**Published:** 2024-08-27

**Authors:** B. D. Ulug, H. C. Ozden

**Affiliations:** ^1^Psychiatry, Hacettepe University, Ankara, Türkiye

## Abstract

**Introduction:**

Comorbidities in addiction: It is a rule rather than an exception. The story starts in childhood; even before, in infancy, may be in utero. The dimensional traits have been already there, existing obviously far before any DSM-5 diagnosis. Developmental qualities of stress sensitivity, impulsivity and emotion dysregulation are the leading ones. Besides, comorbidity research (NESARC being one of the prominents) (Hasin and Grant. Soc Psychiatry Psychiatr Epidem, 2015;50 (11): 1609-1640) addressed childhood abuse, neglect or other childhood adverse experiences as a definite risk factor for adolescence and adult mental disorders, particularly substance use disorders. Developmental and environmental adversities in a mutually amplifying pattern make a vicious cycle in which the individual finally finds an illusionary exit, a pathway to addiction.

**Objectives:**

This presentation aims to discuss the complexities and challenges for the diagnosis and treatment of a patient with a twenty five year follow-up, a survival period for the patient herself as well as for the therapeutic alliance (Ulug, Arch Neuropsychiatr, 2015;52: 213-215).

**Methods:**

Case study: The history and the life chart of her, diagnosed as having at least seven DSM diagnoses, indicate the depth of pyschopathology and the intensity of interventions, most of which failed due to the lack of a transdiagnostic and holistic perspective. A specific focus of the case study will be on the problematic use of Borderline Personality Disorder formulation/diagnosis and its negative, somehow short-coming, impact on the treatment, course and recovery.

**Results:**

The challenges brought by comorbid transdiagnostic cases, similar to the subject of this presentation, have become a common practice for addiction professionals. While big data or empirical large datasets can have their own limitations to help the practitioner for overcoming such challenges, as stated in Stein’s article “it is important to recognize the value of a wide range of complementary research designs including the age-old single-case study, which may sometimes provide clinical insights that outweigh those from big data analyses.” (Stein et al. World Pychiatry, 2022; 21(3): 393-414).

**Conclusions:**

The case study indicates the necessity for transdiagnostic and holistic approach in the management and long term treatment of such difficult-to-diagnose and difficult-to-treat patients.

**Disclosure of Interest:**

None Declared

